# Leaf Senescence, Root Morphology, and Seed Yield of Winter Oilseed Rape (*Brassica napus* L.) at Varying Plant Densities

**DOI:** 10.1155/2017/8581072

**Published:** 2017-08-03

**Authors:** Ming Li, Muhammad Shahbaz Naeem, Shafaqat Ali, Liyan Zhang, Lixin Liu, Ni Ma, Chunlei Zhang

**Affiliations:** ^1^Oil Crops Research Institute, Chinese Academy of Agricultural Science, Key Laboratory of Oil Crop Biology of the Ministry of Agriculture, Key Laboratory of Crop Cultivation and Physiology, Ministry of Agriculture, Wuhan 430062, China; ^2^Department of Agronomy, University of Agriculture, Faisalabad 38000, Pakistan; ^3^Department of Environmental Sciences and Engineering, Government College University, Allama Iqbal Road, Faisalabad 38000, Pakistan; ^4^Key Laboratory of Soil Environment and Pollution Remediation, Institute of Soil Science, Chinese Academy of Sciences, Nanjing 210008, China

## Abstract

In this study, the yield and yield components were studied using a conventional variety Zhongshuang 11 (ZS 11) and a hybrid variety Zhongyouza 12 (ZYZ 12) at varying plant densities. The increase in plant density led to an initial increase in seed yield and pod numbers per unit area, followed by a decrease. The optimal plant density was 58.5 × 10^4^ plants ha^−1^ in both ZS 11 and ZYZ 12. The further researches on physiological traits showed a rapid decrease in the green leaf area index (GLAI) and chlorophyll content and a remarkable increase in malondialdehyde content in high plant density (HPD) population than did the low plant density (LPD) population, which indicated the rapid leaf senescence. However, HPD had higher values in terms of pod area index (PAI), pod photosynthesis, and radiation use efficiency (RUE) after peak anthesis. A significantly higher level of dry matter accumulation and nitrogen utilization efficiency were observed, which resulted in higher yield. HPD resulted in a rapid decrease in root morphological parameters (root length, root tips, root surface area, and root volume). These results suggested that increasing the plant density within a certain range was a promising option for high seed yield in winter rapeseed in China.

## 1. Introduction

Oilseed rape is one of the most important sources of edible oil in the human diet. In recent years, the seed yield has lagged behind the increasing demands driven by population growth. Therefore, the yields of rapeseed crops must be significantly increased [1]. Winter oilseed rape (*Brassica napus* L.) is widely cultivated along the Yangtze River in China, which represents approximately 30% of the total oilseed production worldwide and 89% of the oilseed yield in China [2]. Increasing the seed yield per unit area was an effective approach to promote the fourth leap in the Chinese rapeseed industry [3]. Thus, it is necessary and significant to develop strategies to gain the optimal yield.

Plant density is an important factor affecting seed yield and yield components of oilseed rape [1, 4] and creating a difference between individual and group performance might affect seed yield [5, 6]. Oilseed rape plants have high adaptability to changing environmental conditions [7]; this is also confirmed by Różyło and Pałys (2014) [8]. In European countries with high rapeseed yield, the optimal plant density is approximately 80–150 plants m^−2^ before winter and 60–80 plants m^−2^ at the beginning of spring [9]. However, in China and other semiarid conditions, the transplanting of seedlings has been commonly practiced in oilseed production and a low yield is achieved at a plant density of 10–15 plants m^−2^ [10, 11]. In recent years, the direct seedling with mechanical production is popularizing rapidly, and the modern varieties such as Zhongshuang 11 have the characters of lodging-resistance and high density tolerance [12]. Therefore, we hypothesize that rapeseed yield could be increased by increasing the plant density at a certain range.

Previous studies have demonstrated that photosynthate supply plays an important role in pod and seed development [1, 13–15]. Increasing plant density to a certain degree increases shade to the older leaves lower in the canopy as plant growth progresses, leading to a reduction in canopy light capture [16, 17]. A reduction in the light intensity below the light compensation point leads to a negative carbon balance, which triggers senescence, resulting in the death or falling of leaves [16, 18–20]. Chlorophyll degradation and malondialdehyde content were also characterized during leaf senescence [18, 21, 22]. Nevertheless, plants increase the pod wall area index by allocating assimilates and nitrogen to developing pods [23]. With increasing plant density, the competition for growing space increases [24], particularly the competition for the absorption of water and nutrients, thereby restraining growth and decreasing the final yield of individual plants [25, 26]. Belowground competition often reduces plant performance more than aboveground competition [27]. The plant root is a vital organ for crops to absorb water and nutrients [28]. It has been reported that increased root growth might lead to the increased extraction of water from the soil, but this advantage might be more than offset by a decline in the harvest index because there is less assimilate available for grain growth [29]. Therefore, understanding the root morphology of winter rapeseed at varying plant densities might provide information concerning the pivotal mechanism for the stagnation of oilseed rape yield on farms and benefit crop management for the desirable utilization of nutrients during the photosynthetic period.

In winter oilseed rape, the leaves are the main photosynthetic source before anthesis, whereas the lower part of the plant canopy becomes part of the source after anthesis, and during pod development, the photosynthetic rate from green pods during seed filling contributes to approximately 2/3 of the total seed weight [30]. There have been many documents that investigated the yield and yield components of winter rapeseed, but few studies have investigated the leaf-pod physiology and the root growth response to the plant densities. The objectives of the present study were to (i) optimize the plant density of winter oilseed rape in the center of the Yangtze River basin in the modern cultivation system in China and (ii) determine the leaf-pod growth, root morphology, and the potential physiological characteristics for high seed yield.

## 2. Materials and Methods

### 2.1. Experimental Site

The field trials were conducted from 2010 to 2014 at Yangluo Experimental Station of the Oil Crops Research Institute in Wuhan, Hubei, China (30°6′N, 114°1′E), which is located approximately in the center of the Yangtze River basin. This area is characterized by yellow-brown soil in the experimental field. The surface soil (0–30 cm) was sampled at the beginning of each growing season. The soil samples were air dried, ground, and analyzed for pH value, dissolved organic carbon (DOC), total nitrogen, alkaline digested N, available phosphorus, available potassium, and available boron contents (Table 1). The soil agrochemical characteristics were described according to Wang et al. in 2010 [31].

### 2.2. Experimental Design

The first experiment was conducted during the 2010-2011 and 2011-2012 growing seasons to evaluate the effects of plant density on seed yield and yield components. Conventional winter rapeseed variety Zhongshuang 11 (ZS 11) and the hybrid variety Zhongyouza 12 (ZYZ 12), two elite winter rapeseed varieties commonly grown in the Yangtze River basin, were used. The seeds were sown on 28 September in both 2010 and 2011. A split-plot design was used with three replicates. The main plots comprised five plant densities (27.0 × 10^4^, 37.5 × 10^4^, 48.0 × 10^4^, 58.5 × 10^4^, and 69.0 × 10^4^ plants ha^−1^), and the subplots comprised two varieties. Each subplot was 2 × 10 m, with rows approximately 30–35 cm apart (three rows per meter). The plants were finalized by hand when the seedlings had fully developed 4-5 true leaves, and the spaces between seedlings ranged from 4 to 11 cm to achieve different planting densities. Each plot was fertilized at the average fertilizer level in the Yangtze River basin with urea (195 kg N ha^−1^), superphosphate (75 kg P_2_O_5_ ha^−1^), potassium chloride (105 kg K_2_O ha^−1^), and borax (9 kg boron ha^−1^). Approximately 60% of the nitrogen fertilizer was applied at sowing and the remaining 40% of the nitrogen fertilizer was applied at the seedling stage, whereas phosphorus, potassium, and borax were all applied at sowing.

The second experiment was conducted during the 2012-2013 and 2013-2014 seasons to study physiological traits of different populations. In the first experiment, low seed yield was obtained at a plant density of 27.0 × 10^4^ plant ha^−1^, referred to as the low plant density (LPD) population. The highest yield was obtained at a plant density of 58.5 × 10^4^ plant ha^−1^, referred to as the high plant density (HPD) population for both varieties. The second experiment was a randomized complete block design with three replicates. The seeds were sown on 28 September in both 2012 and 2013. The plot area was 10 m long × 2 m wide and comprised 30 rows. A 1 m border surrounded each plot. The application rates of N, P_2_O_5_, and K_2_O were the same as those used in the 2010-2011 and 2011-2012 growing seasons.

### 2.3. Yield and Yield Components

In 2010–2014 growing seasons, at maturity, plants per unit area (m^2^) were sampled, and the yield components (i.e., pods per unit area, seeds per pod, and 1000-seed weight) at each plot were determined. Seed yield was determined by harvesting the plants of 5 m^2^ area in each plot, and the seed yields per unit area (ha^−1^) were calculated, with 9% standard moisture content.

### 2.4. Determination of Chlorophyll and Malondialdehyde Contents

In 2012-2013 and 2013-2014 seasons, the chlorophyll and malondialdehyde contents in the leaves of HPD population and LPD population were determined. The frozen leaves (0.2 g) were first ground to a fine powder in liquid nitrogen, and chlorophyll was extracted after immersing the powder with cold acetone overnight at 4°C. The supernatant containing chlorophyll was generated after centrifugation at 10,000 ×g for 30 min. The residue was washed several times with cold acetone until it became colorless. The pooled supernatant was diluted to 10 mL with acetone until the final acetone concentration was 80%. The chlorophyll content per fresh weight of leaves was calculated as previously described [32]. The MDA content was measured according to Liu et al. (2006) [33], with modifications. Briefly, the frozen leaf samples (0.5 g) were homogenized in 4 mL of 0.05 M phosphate buffer (pH 7.8) and centrifuged for 15 min at 10,000 ×g. The supernatant was collected, and 1 mL of the supernatant was mixed with 3 mL of 0.5% thiobarbituric acid. Subsequently, the mixture was boiled for 15 min, followed by quick cooling in an ice bath and centrifugation at 12,000 ×g for 15 min. Subsequently, the supernatant was collected, and the absorbance was measured at 450, 532, and 600 nm (*A*_450_, *A*_532_, and *A*_600_). The MDA content was calculated according to the following formula: 6.453 × (*A*_532_ − *A*_600_) − 0.563 × *A*_450_.

### 2.5. Green Leaf Area Index (GLAI), Pod Area Index (PAI), Photosynthesis, and Radiation Use Efficiency (RUE)

In 2012-2013 and 2013-2014 seasons, the green leaf area was measured by passing the leaves through a LI-3100 leaf area meter (LiCor, Lincoln, NE, USA) at 7-day intervals after peak anthesis. The gas exchange analysis was conducted in the LPD and HPD populations of two varieties using a Portable Photosynthesis System (LI-6400; LiCor) on the leaves from 09:00 to 11:00. The net photosynthetic rates (Pn), stomatal conductance (Gs), intercellular CO_2_ concentration (Ci), and transpiration rate (Tr) were determined. The data were collected automatically every 2-3 min with 10 replications for every plot.

At the seed-filling stage, fifty pods on the main inflorescences and all of the branches were randomly sampled to measure the pod length and width, and the pod wall area was calculated according to Clarke (1978) [34]. The pod photosynthesis was measured along with the pod sampling. The GLAI and PAI were then determined on a ground area basis.

Canopy radiation interception was measured at 7-day intervals from flowering stage to maturity using SunScan Canopy Analysis System (Delta-T Devices Ltd., UK). To measure the transmitted radiation, the 1 m probe was placed perpendicular to rows near soil surface for each plot. Another sensor (model BF5) was located outside the canopy for measurement of incident photosynthetically active radiation (PAR) [35, 36]. Measuring was completed within 1.5 h of solar noon on clear days. Five positions were randomly selected and marked in each plot for measuring canopy radiation interception. Canopy radiation interception was calculated as the percentage of incoming radiation intensity that was intercepted by the canopy [100 × (incoming radiation intensity − radiation intensity inside canopy)/incoming radiation intensity] [36, 37]. Intercepted radiation was calculated using the average canopy radiation interception and accumulated incoming solar radiation during the target growth period [1/2 × (canopy radiation interception at the beginning of the growth period + canopy light interception at the end of the growth period) × accumulated incoming radiation during the growth period] [36, 37]. At maturity, the plants per unit area (m^2^) in each plot were randomly selected, and the aerial parts were collected. The aerial parts were separated into stems, pod walls, and seeds and air dried for approximately 1 month to record dry biomass. The RUE was calculated as the ratio of above ground total dry weight at maturity to intercepted radiation during the flowering stage to maturity [37].

### 2.6. Nitrogen Utilization Efficiency

Different aerial organs at maturity at different densities were ground into powder, and an appropriate amount of plant material was used to determine the total nitrogen content using a modified Kjeldahl digestion method [38]. The nitrogen utilization efficiency and related parameters were calculated using the following equations, with some modification [9, 39, 40]:

(1) Total  nitrogen  uptake g m−2=dry  matter  of  stems×nitrogen  content  of  stems+dry  matter  of  pod  wall×nitrogen  content  of  pod  wall+seed  yield×nitrogen  content  of  seedsNitrogen  utilization  efficiency kg kg−1=seed  yieldtotal  nitrogen  uptakeNitrogen  harvest  index %=seed  yield×nitrogen  content  of  seedstotal  nitrogen  uptake×100.

### 2.7. Measurement of Root Morphology

Root digging was performed according to Majdi (1996) [41]. The dynamic sampling time points were 0, 7, 14, 21, and 28 DAPA. The sampling area in each plot was 0.5 m long × 0.5 m wide × 1.0 m high. Before measuring the root morphology, the leaves and pods of the plants were removed, and the stems were cut at 0.4 m above the ground to avoid obstruction of the aerial parts with neighboring plants [42]. The roots in the square were carefully collected, and the root length, number of root tips, root surface area, and root volume per unit area were scanned and analyzed using the WinRHIZO 2009 software (Regent Company, Canada).

### 2.8. Data Analysis

We performed multiway ANOVA with critical values of *p* = 0.05 using the Statistix 8 software. Significant pairwise differences between the mean values were identified using Duncan's multiple range tests (*p* < 0.05) in SPSS software (version 16.0; SPSS Inc., Chicago, IL, USA). The mean values were separated using Duncan's multiple range test. All statistical determinations were made at *p* = 0.05. Correlations between the seed yield and pod numbers per unit area, seeds per pod, and 1000-seed weight in different plant densities were analyzed in 2010–2012 growing seasons, and a correlation analysis was performed to determine the relationships between seed yield and dry matter weight, radiation use efficiency, nitrogen utilization efficiency, and nitrogen harvest index in 2012–2014 seasons [43].

## 3. Results

### 3.1. Yield and Yield Components

In the first experiment, which was conducted during the 2010-2011 and 2011-2012 growing seasons, the pod numbers per unit area were initially positively and then negatively affected after increasing plant density. Compared with a density of 27.0 × 10^4^ plant ha^−1^, the pod numbers per unit area of ZS 11 and ZYZ 12 varieties increased significantly at 48.0 × 10^4^ plant ha^−1^ and 58.5 × 10^4^ plant ha^−1^, respectively, and the maximum pod numbers per unit area were obtained at a plant density of 58.5 × 10^4^ plant ha^−1^ for both varieties (Table 2).

Experimental treatments had pronounced effects on seeds per pod. The seeds per pod of the two varieties were significantly decreased with increasing plant densities, but the 1000-seed weight showed no significant differences at the examined plant densities. The ANOVA results showed that the pod numbers and seeds per pod were obviously affected not only by the year, variety, and plant density but also by plant interactions, whereas the 1000-seed weight was not significantly affected.

**Figure 1:**
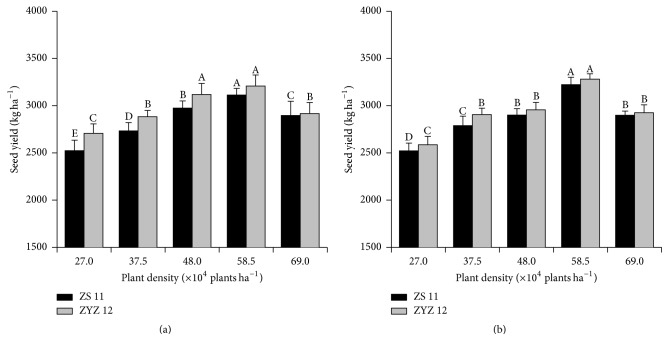
Seed yield per unit area (kg ha^−1^) at various plant densities during the 2010-2011 and 2011-2012 growing seasons. (a) Seed yield per unit area (kg ha^−1^) at various plant densities during the 2010-2011. (b) Seed yield per unit area (kg ha^−1^) at various plant densities during the 2011-2012. Different letters indicate significant differences at *p* < 0.05 between treatment groups according to Duncan's test.

The seed yields per unit area were also initially positively and then negatively affected with increasing plant density during the 2010-2011 and 2011-2012 growing seasons (Figure 1).

**Figure 2:**
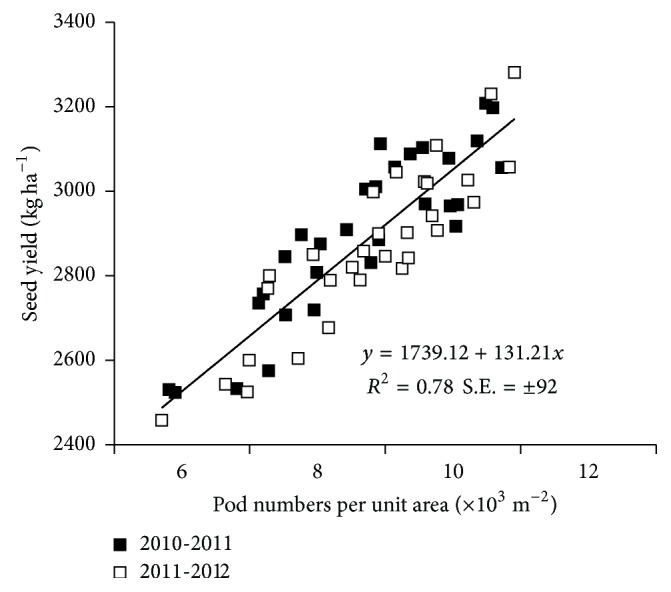
Regression of seed yield (kg ha^−1^) over pod numbers per unit area in the two varieties across the 2010-2011 and 2011-2012 growing seasons.

The highest and lowest values of the seed yields per plot were obtained at 58.5 × 10^4^ and 27.0 × 10^4^ plant ha^−1^ during the 2010-2011 and 2011-2012 growing seasons, respectively. Compared with 27.0 × 10^4^ plant ha^−1^, the seed yields per unit area at 58.5 × 10^4^ plant ha^−1^ for ZS 11 and ZYZ 12 significantly increased 23.3% and 18.5%, respectively, during the 2010-2011 season and 27.6% and 26.7%, respectively, during the 2011-2012 season. In addition, the pod numbers per unit area displayed a strong correlation with seed yield (*R*^2^ = 0.78) (Figure 2), whereas the number of seeds per pod and the 1000-seed weight showed an insignificant correlation with seed yield (*R*^2^ = 0.16, *R*^2^ < 0.01, resp.).

### 3.2. Chlorophyll and Malondialdehyde Contents

**Figure 3:**
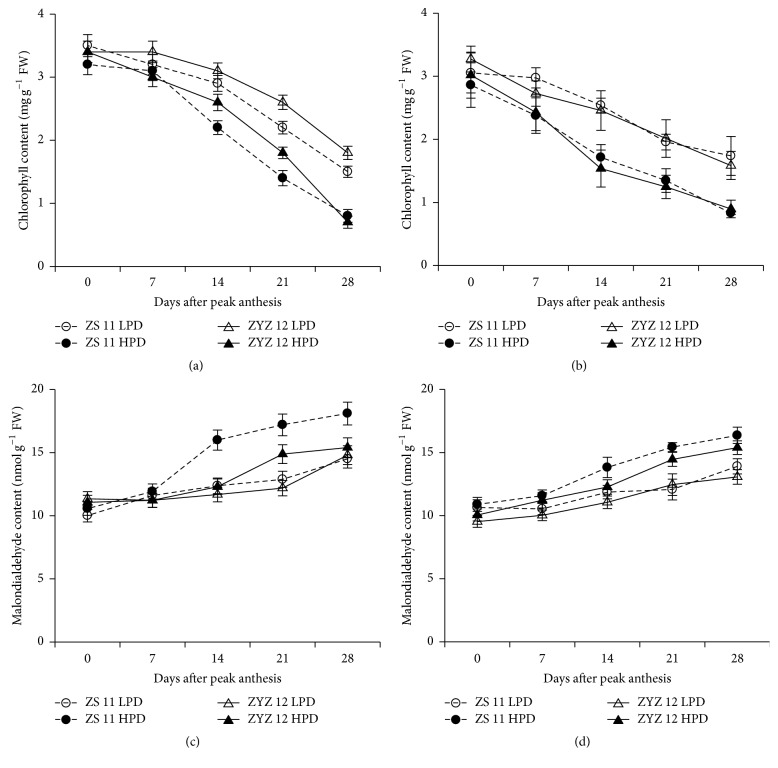
Leaf chlorophyll and malondialdehyde contents in the low plant density (LPD) population and high plant density (HPD) population in ZS 11 and ZYZ 12 at 7-day intervals after peak anthesis during the 2012-2013 and 2013-2014 growing seasons. (a, b) Leaf chlorophyll in LPD and HPD in ZS 11 and ZYZ 12 at 7-day intervals after peak anthesis during the 2012-2013 and 2013-2014 growing seasons. (c, d) Malondialdehyde contents in LPD and HPD in ZS 11 and ZYZ 12 at 7-day intervals after peak anthesis during the 2012-2013 and 2013-2014 growing seasons. The bars indicate the SD.

The chlorophyll content in the leaves decreased more rapidly in HPD than in LPD, and HPD showed a lower chlorophyll content (Figures 3(a) and 3(b)). However, the MDA content in LPD and HPD leaves increased, and the MDA content in HPD leaves was much higher and increased more rapidly at 7 DAPA (Figures 3(c) and 3(d)) in 2012-2013 and 2013-2014 growing seasons.

### 3.3. Green Leaf Area Index, Pod Area Index, Photosynthesis, and Radiation Use Efficiency

**Figure 4:**
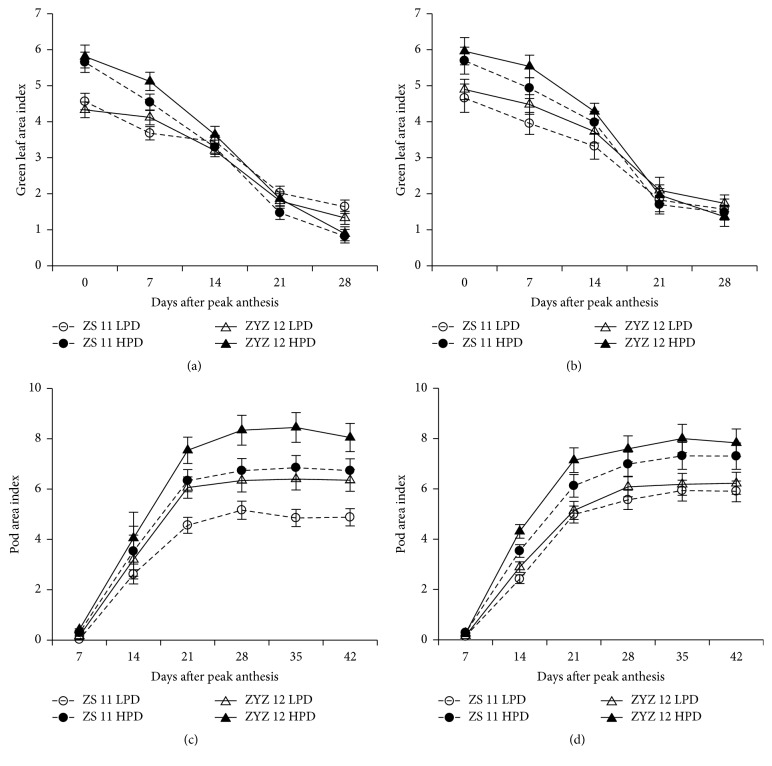
Green leaf area index (GLAI) and pod area index (PAI) in the low plan density (LPD) population and high plant density (HPD) population in ZS 11 and ZYZ 12 at 7-day intervals after peak anthesis during the 2012-2013 and 2013-2014 growing seasons. (a, b) GLAI in LPD and HPD in ZS 11 and ZYZ 12 at 7-day intervals after peak anthesis during the 2012-2013 and 2013-2014 growing seasons. (c, d) PAI in LPD and HPD in ZS 11 and ZYZ 12 at 7-day intervals after peak anthesis during the 2012-2013 and 2013-2014 growing seasons. The bars indicate the SD.

Figure 4 showed the dynamics of the green leaf area index (GLAI) and pod area index (PAI) after peak anthesis of the two varieties during the 2012-2013 and 2013-2014 growing seasons.

The GLAI in both HPD and LPD rapidly decreased after peak anthesis, and this value was lower in HPD than in LPD after 14 DAPA. During the 2012-2013 season, the GLAI from 0 to 28 days after peak anthesis in HPD decreased 85.5% and 84.7% in ZS 11 and ZYZ 12, respectively, whereas the GLAI in LPD decreased 64.0% and 69.3% in ZS 11 and in ZYZ 12, respectively (Figure 4(a)). During the 2013-2014 season, the GLAI in HPD decreased 74.0% and 77.2% in ZS 11 and ZYZ 12, respectively, whereas the GLAI in LPD decreased 66.2% and 64.7% in ZS 11 and ZYZ 12, respectively (Figure 4(b)). The pod area index in all of the populations increased rapidly from 0 to 21 days after peak anthesis and reached a maximum at 28 to 42 days after peak anthesis. A higher pod area was observed in HPD as compared to LPD irrelative of varieties and growing season (Figures 4(c) and 4(d)).

**Figure 5:**
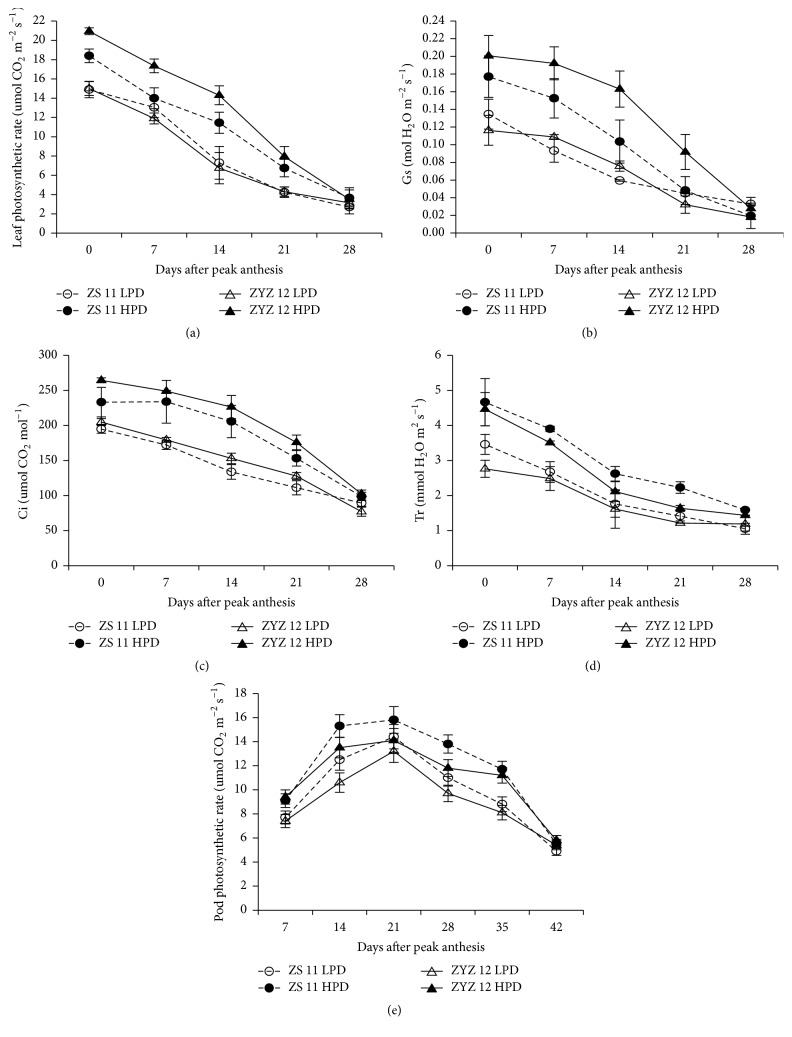
The leaf photosynthetic rate (Pn), stomatal conductance (Gs), intercellular CO_2_ concentration (Ci), transpiration rate (Tr), and pod photosynthetic rate in the low plan density (LPD) population and high plant density (HPD) population in ZS11 and ZYZ 12 at 7-day intervals after peak anthesis during the 2012-2013 growing seasons. (a) Pn, (b) Gs, (c) Ci, (d) Tr, and (e) pod photosynthetic rate in LPD and HPD populations in ZS11 and ZYZ12 at 7-day intervals after peak anthesis during the 2012-2013 growing seasons. The bars indicate the SD.

**Figure 6:**
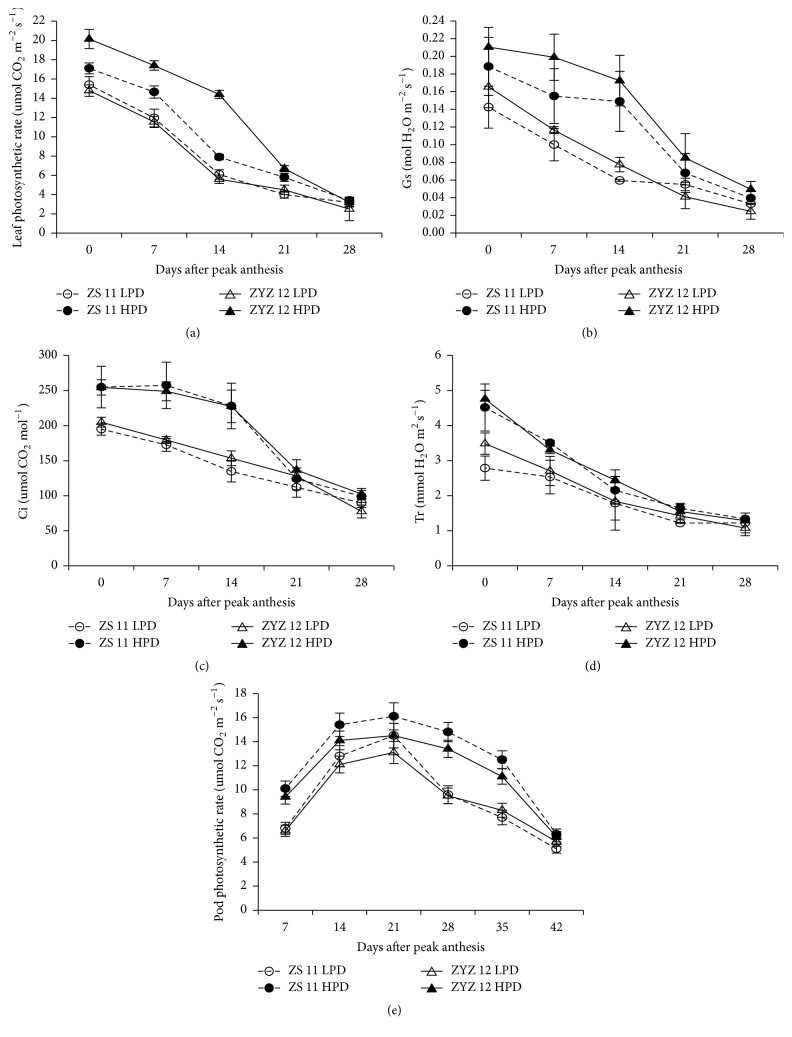
The leaf photosynthetic rate (Pn), stomatal conductance (Gs), intercellular CO_2_ concentration (Ci), transpiration rate (Tr), and pod photosynthetic rate in the low plan density (LPD) population and high plant density (HPD) population in ZS11 and ZYZ 12 at 7-day intervals after peak anthesis during the 2013-2014 growing seasons. (a) Pn, (b), Gs (c), Ci (d), Tr, and (e) pod photosynthetic rate in LPD and HPD populations in ZS11 and ZYZ12 at 7-day intervals after peak anthesis during the 2013-2014 growing seasons. The bars indicate the SD.

In both seasons, the photosynthetic rate (Pn), stomatal conductance (Gs), intercellular CO_2_ concentration (Ci), and transpiration rate (Tr) of leaves decreased rapidly after flowering, whereas the values declined rapidly in the high-yield population 21 days after peak anthesis (Figures 5(a)*–*5(d) and 6(a)*–*6(d)). The pod photosynthesis increased rapidly from 7 to 21 days and reached a maximum ~21 days after peak anthesis. The change trend of pod photosynthetic rate also showed that the high-yield populations had a longer duration of high photosynthetic rates from 14 to 28 DAPA in both varieties (Figures 5(e) and 6(e)).

High plant density population had slightly lower accumulated incident radiation than the low plant density population owing to their shorter growth duration in two growing seasons in two varieties (Table 3).

However, the intercepted radiation was significantly higher in HPD population corresponding to their LPD populations. It is interesting that the higher canopy radiation interception in HPD population was due to the higher PAI. Compared with LPD, the dry matter weight in HPD increased 42.3% and 47.4% in ZS 11 during the 2012-2013 and 2013-2014 seasons, respectively, and the dry matter weight in ZYZ 12 increased 46.7% and 55.1%, respectively. In 2012-2013 and 2013-2014 seasons, HPD population had 18.7% and 24.3% higher RUE than LPD population in ZS 11, respectively. HPD population had 20.5% and 38.1% higher RUE than LPD population in ZYZ 12, respectively. The ANOVA results showed that the incident radiation, intercepted radiation, intercepted percent, total dry weight, and radiation use efficiency were all significantly or extremely significantly affected not only by the year, variety, and plant density but also by plant interactions.

### 3.4. Nitrogen Utilization Efficiency

**Figure 7:**
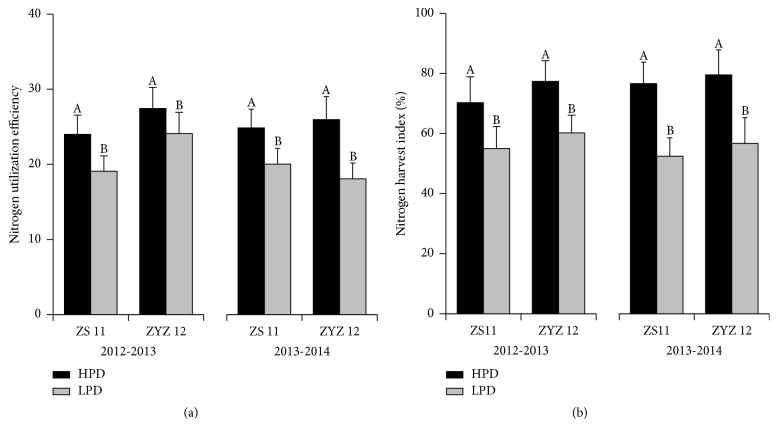
Nitrogen utilization efficiency and nitrogen harvest index (NHI) in the low plant density (LPD) population and high plant density (HPD) population in ZS 11 and ZYZ 12 during the 2012-2013 and 2013-2014 growing seasons. (a) Nitrogen utilization efficiency in LPD and HPD in ZS 11 and ZYZ 12 during the 2012-2013 and 2013-2014 growing seasons. (b) NHI in LPD and HPD in ZS 11 and ZYZ 12 during the 2012-2013 and 2013-2014 growing seasons. Different letters indicate significant differences at *p* < 0.05 between treatment groups according to Duncan's test.

The nitrogen utilization efficiency increased 25.8% and 24.2% in HPD of ZS 11, respectively, and 14.0% and 43.7% in HPD of ZYZ 12 during the 2012-2013 and 2013-2014 seasons, respectively (Figure 7(a)). The nitrogen harvest index in HPD was 27.8% and 46.2% higher in ZS11 and it was 28.5% and 40.2% higher in ZYZ 12 during the two growing seasons, respectively (Figure 7(b)).

**Figure 8:**
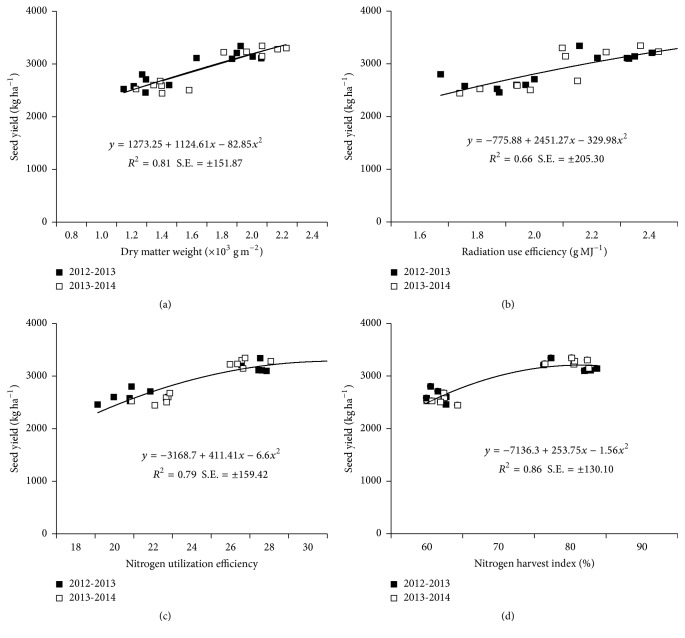
Regression of seed yield (kg ha^−1^) over dry matter weight, radiation use efficiency, nitrogen utilization efficiency, and nitrogen harvest index in the two varieties across the 2012-2013 and 2013-2014 growing seasons. (a) Regression of seed yield (kg ha^−1^) over dry matter weight in the two varieties across the 2012-2013 and 2013-2014 growing seasons. (b) Regression of seed yield (kg ha^−1^) over radiation use efficiency in the two varieties across the 2012-2013 and 2013-2014 growing seasons. (c) Regression of seed yield (kg ha^−1^) over nitrogen utilization efficiency in the two varieties across the 2012-2013 and 2013-2014 growing seasons. (d) Regression of seed yield (kg ha^−1^) over nitrogen harvest index in the two varieties across the 2012-2013 and 2013-2014 growing seasons.

The dry matter weight, radiation use efficiency, nitrogen utilization efficiency, and nitrogen harvest index were significantly correlated with seed yield (*R*^2^ = 0.83, *R*^2^ = 0.66, *R*^2^ = 0.79, and *R*^2^ = 0.86, resp.) (Figure 8).

### 3.5. Root Morphology After Peak Anthesis

For both HPD and LPD populations in the two varieties, the root length, root tips, root surface area, and root volume per unit area declined after peak anthesis (Table 4), but the data suggested that HPD had a larger reduction than LPD in all root characteristics.

During the 2012-2013 season, these values decreased by 78.3%, 43.9%, 64.5%, and 45.3%, respectively, at 28 DAPA compared with 0 DAPA in HPD of ZS 11, whereas a decrease of 68.9%, 41.9%, 61.5%, and 37.8%, respectively, was observed in LPD. The root parameters showed a decrease of 64.6%, 54.3%, 67.6%, and 49.5%, respectively, in HPD of ZYZ 12, whereas a decrease of 62.5%, 41.3%, 53.6%, and 39.0%, respectively, was observed in LPD. Similarly, during the 2013-2014 season, these root values at 28 DAPA decreased by 77.8%, 46.9%, 54.8%, and 43.8%, respectively, in HPD of ZS 11, but a lower reduction of 67.0%, 37.8%, 50.0%, and 42.4%, respectively, was observed in LPD. In HPD of ZYZ 12, these values decreased by 64.5%, 49.0%, 54.5%, and 52.1%, respectively, and a lower reduction of 55.7%, 40.2%, 48.1%, and 51.0%, respectively, was observed in LPD. The ANOVA results showed that the year, variety, and DAPA significantly affected the root length, root tips, root surface area, and root volume. Additionally, the interactions among these factors significantly affect root morphology.

## 4. Discussion

In the present study, the seed yield of winter oilseed rape can be effectively increased by increasing plant density from 27.0 × 10^4^ plant ha^−1^ to 58.5 × 10^4^ plant ha^−1^ and decreases at the plant density of 69.0 × 10^4^ plant ha^−1^. It is consistent that an increasing the number of plants per unit area is associated with a better use of arable land and better light interception, but this does not always result in higher yielding capacity [11, 44–50]. The yield of winter oilseed rape might be a function of the pod numbers per unit area [51], the number of seeds per pod, and the 1000-seed weight. With increasing plant density, the pod numbers per unit area significantly increase and then decrease. Plant density also shows an apparent negative effect on seeds per pod but does not significantly influence the 1000-seed weight. Therefore, the increase in seed yield primarily reflected the optimization of pod numbers per unit area [13, 47]. Interestingly, the seeds per unit area showed a significantly positive correlation with the seed yield (data not shown), although the seeds per pod decreased with the increasing plant density. Indeed, a previous study demonstrated that there was still much space for improving seeds per unit area at high plant density to increase the seed yield [13]. Oilseed rape (*Brassica napus* L.) is a crop with a complex aerial architecture that determines a light gradient over the foliage [17]. As reported, the leaf is the photosynthetic source before anthesis, and the green pod has both the source-sink function after anthesis [30]. In the present study, chlorophyll degradation and MDA content increased, which triggered the leaf senescence [21] and resulted in the rapidly decrease of GLAI and leaf photosynthesis in HPD population in the two varieties after anthesis. However, the PAI and pod photosynthesis increased rapidly, suggesting that leaf senescence was increased concomitantly with the formation of efficient pod canopy. In other words, the successful alteration of architecture and function between leaf and pod happened in HPD population after anthesis. It was postulated that the HPD contributed to upright branches, and the pods profile compactly arranged so that these pods could not shade over each other. This phenotype improved the pods to acquire the light energy and resulted in higher canopy radiation interception and radiation use efficiency. The results were in agreement with Morrison and Stewart (1995) [52] and Wang et al. (2015) [36], who suggested that plant density had great effect on the radiation use efficiency, and the positive correlation between radiation efficiency and seed yield confirmed the results from Katsura et al. (2007) [53], which highlighted the fact that the radiation use efficiency might be the main factor in the high grain yield of rice. Thus, the high yield at HPD might reflect the harmonious alteration between leaf senescence and better light interception within the pod canopy [23].

Previous studies have also indicated that high yield is associated with dry matter production [37, 53]. The high dry matter accumulation was due to the good balance of source-sink and the reserved carbohydrates in the vegetative tissues contributed significantly to the final seed yield [1, 54]. Therefore, it is necessary to produce sufficient vegetative biomass to support seed filling [55, 56]. Furthermore, N reallocation from lower leaves, which is also associated with leaf senescence, contributed to the development of pods and seeds [16]. The HPD population had higher LAI at peak anthesis and then decreased rapidly, which suggested that the C and N compounds remobilized more efficiently toward growing points under shading treatment [17]. High NHI represented the increased capacity of the genotype to mobilize and transform nitrogen from the leaves and culms to the pods and seeds [39]. In the present study, higher nitrogen utilization and NHI in high plant density population showed more N translocation from senescent leaves to reproductive organs, which benefit the high seed yield. Prior to this study, little information was available describing the changes in root morphology and physiology at different plant densities. Roots are vital organs for yield improvement. Increasing plant density could increase the intraspecific competition for water and mineral nutrients [27]. The functional performance of the roots is closely associated with root morphological characteristics [57]. In the present study, the root parameters decreased rapidly at high plant density, which can supply more water and nutrient for the shoot, improve the PAI, photosynthetic rate, and longer duration of high photosynthesis (data not shown), and make the contribution to the shoot growth and seed filling [58, 59].

## 5. Conclusions

The effects of five different plant densities were examined to optimize the population under modern cultivation systems and clarify the mechanism of high seed yield. The results indicated that a higher seed yield and optimal plant density were obtained after increasing the plant density to a certain range. In high plant density population, it showed a rapid decrease in GLAI and chlorophyll content as well as the rapid increase of MDA content after peak anthesis. The high yield highlighted the rapid increase of PAI and pod photosynthesis concomitant with accelerated leaf senescence after peak anthesis. The higher reduction in root morphological parameters, namely, root length, root tips, root surface area, and root volume, the higher accumulation in dry biomass, and higher N utilization efficiency in higher plant density treatment at peak anthesis suggested that that higher nutrient concentration could be available for shoot growth and seed filling.

## Figures and Tables

**Table 1:** Soil properties measured at the beginning of each growing season from 2010 to 2014.

Parameter	Unit	2010-2011	2011-2012	2012-2013	2013-2014
pH		6.65	6.82	6.70	6.91
Dissolved organic carbon	mg kg^−1^	85.3	95.1	104.1	100.6
Total N	g kg^−1^	1.51	1.69	1.79	1.56
Alkaline digested N	mg kg^−1^	74.2	78.2	79.3	77.8
Available phosphorus	mg kg^−1^	46.2	45.7	50.5	49.7
Available potassium	mg kg^−1^	63.1	60.3	68.4	65.4
Available boron	mg kg^−1^	0.45	0.51	0.66	0.63

**Table 2:** Yield components of ZS 11 and ZYZ 12 populations at varying plant densities during the 2010-2011 and 2011-2012 growing seasons.

Year	Variety	Plant density (×10^4^ plants ha^−1^)	Pod numbers per unit area (×10^3^ m^−2^)	Seeds per pod	1000-seed weight (g)
2010-2011	ZS 11	27.0	5.81^e^	18.34^a^	3.89^a^
		37.5	7.20^d^	16.89^b^	3.81^a^
		48.0	7.99^b^	16.83^b^	3.57^a^
		58.5	8.86^a^	16.28^b^	3.68^a^
		69.0	7.52^c^	15.73^c^	3.55^a^
	ZYZ 12	27.0	7.28^d^	19.36^a^	3.83^a^
		37.5	8.71^c^	18.54^b^	3.79^a^
		48.0	9.94^a^	17.84^c^	3.60^a^
		58.5	10.07^a^	17.47^c^	3.68^a^
		69.0	9.59^b^	16.62^d^	3.56^a^

2011-2012	ZS 11	27.0	6.81^c^	19.69^a^	3.97^a^
		37.5	8.43^b^	19.50^a^	3.84^a^
		48.0	9.14^a^	18.25^b^	3.77^a^
		58.5	9.37^a^	17.46^c^	3.63^a^
		69.0	8.79^b^	17.51^c^	3.52^a^
	ZYZ 12	27.0	7.95^d^	20.22^a^	3.76^a^
		37.5	9.55^c^	19.54^b^	3.52^a^
		48.0	10.59^a^	18.82^c^	3.53^a^
		58.5	10.72^a^	17.81^d^	3.46^a^
		69.0	9.96^b^	17.39^d^	3.30^a^

Year (Y)			*∗∗*	*∗∗*	^†^NS
Variety (V)			*∗∗*	*∗*	^†^NS
Year (Y) × variety (V)			*∗∗*	*∗∗*	^†^NS

*∗* indicates statistical significance of the correlation coefficients at *p* < 0.05; *∗∗* indicates statistical significance of the correlation coefficients at *p* < 0.01; † NS indicates no significance of the correlation coefficients at *p* < 0.05; the mean values in a column with different letters indicate significant differences at *p* < 0.05 between treatment groups according to Duncan's multiple range test.

**Table 3:** Radiation use efficiency and its related parameters for low plant density (LPD) population and high plant density (HPD) population in ZS 11 and ZYZ 12 from flowering to maturity during 2012-2013 and 2013-2014 growing seasons.

Sowing date (month/day)	Plant density (×10^4^ plants ha^−1^)	Incident radiation (MJ m^−2^)	Intercepted radiation (MJ m^−2^)	Intercepted percent (%)	Total dry weight (g m^−2^)	Radiation use efficiency (g MJ^−1^)
2012-2013						
ZS 11	LPD	942^a^	612^b^	65.0^b^	1146.7^b^	1.87^b^
	HPD	885^b^	735^a^	83.1^a^	1631.2^a^	2.22^a^
ZYZ 12	LPD	867^a^	648^b^	74.7^b^	1295.0^b^	2.00^b^
	HPD	839^b^	787^a^	93.8^a^	1899.2^a^	2.41^a^

2013-2014						
ZS 11	LPD	977^a^	681^b^	69.7^b^	1229.3^b^	1.81^b^
	HPD	924^b^	804^a^	87.1^a^	1812.6^a^	2.25^a^
ZYZ 12	LPD	882^a^	722^b^	81.9^b^	1399.3^b^	1.94^b^
	HPD	869^a^	809^a^	93.1^a^	2170.2^a^	2.68^a^

Year (Y)		*∗*	*∗∗*	*∗*	*∗∗*	*∗∗*
Variety (V)		*∗*	*∗∗*	*∗∗*	*∗∗*	*∗∗*
Year (Y) × variety (V)		*∗*	*∗∗*	*∗∗*	*∗∗*	*∗∗*
Year (Y) × density (D)		*∗*	*∗∗*	*∗∗*	*∗∗*	*∗∗*
V × D		*∗*	*∗∗*	*∗∗*	*∗∗*	*∗∗*
Y × V × D		*∗*	*∗∗*	*∗∗*	*∗∗*	*∗∗*

*∗* indicates statistical significance of the correlation coefficients at *p* < 0.05; *∗∗* indicates statistical significance of the correlation coefficients at *p* < 0.01; the mean values in a column with different letters indicate significant differences at *p* < 0.05 between treatment groups according to Duncan's multiple range test.

**Table 4:** Root length, number of root tips, root surface area, and root volume per unit area (0.25 m^3^) for low plant density population (LPD) and high plant density population (HPD) in ZS 11 and ZYZ 12 at 7-day intervals after peak anthesis during the 2012-2013 and 2013-2014 growing seasons.

Year	Variety	Day after peak anthesis (DAPA)	Root length (m)		Root tips (×10^3^)		Root surface area (m^2^)		Root volume (dm^3^)	
			LPD	HPD	LPD	HPD	LPD	HPD	LPD	HPD
2012-2013	ZS 11	0	14.27^a^	25.58^a^	2.98^a^	3.62^a^	0.26^a^	0.31^a^	4.54^a^	5.52^a^
		7	9.75^b^	16.38^b^	2.63^b^	3.26^a^	0.22^b^	0.26^b^	3.69^b^	5.12^a^
		14	7.52^c^	8.84^c^	2.07^c^	2.28^b^	0.18^c^	0.25^b^	3.82^b^	4.38^b^
		21	6.12^c^	6.11^d^	2.04^c^	2.03^c^	0.15^c^	0.11^c^	2.92^c^	3.70^c^
		28	4.44^d^	5.55^d^	1.73^d^	2.02^c^	0.10^d^	0.11^c^	2.83^c^	3.02^d^
	ZYZ 12	0	13.29^a^	16.17^a^	2.69^a^	3.72^a^	0.28^a^	0.34^a^	5.02^a^	5.23^a^
		7	9.95^b^	14.54^a^	2.62^a^	3.39^a^	0.22^b^	0.25^b^	4.32^b^	5.98^a^
		14	6.50^c^	6.68^b^	1.83^b^	2.18^b^	0.16^c^	0.20^b^	3.48^c^	3.04^b^
		21	6.27^c^	5.82^c^	1.68^b^	1.78^c^	0.15^c^	0.13^c^	2.95^d^	2.59^c^
		28	4.99^d^	5.72^c^	1.58^b^	1.70^c^	0.13^d^	0.11^c^	2.06^d^	2.64^c^

2013-2014	ZS11	0	13.83^a^	25.91^a^	2.88^a^	3.52^a^	0.26^a^	0.31^a^	4.93^a^	5.40^a^
		7	10.65^b^	15.54^b^	2.80^a^	3.40^a^	0.22^a^	0.28^a^	4.25^b^	5.28^a^
		14	7.73^c^	8.74^c^	1.96^b^	2.22^b^	0.17^b^	0.22^b^	3.96^bc^	4.48^b^
		21	5.43^c^	6.32^d^	1.87^b^	2.10^b^	0.15^b^	0.16^c^	3.13^c^	3.56^c^
		28	4.56^d^	5.74^d^	1.79^b^	1.87^c^	0.13^c^	0.14^c^	2.84^d^	2.38^d^
	ZYZ12	0	12.57^a^	16.01^a^	2.81^a^	3.65^a^	0.27^a^	0.33^a^	4.99^a^	5.56^a^
		7	11.37^a^	14.78^a^	2.49^b^	3.50^a^	0.24^a^	0.25^b^	4.50^a^	5.78^a^
		14	5.80^b^	6.17^b^	1.80^c^	2.04^b^	0.15^b^	0.17^c^	3.19^b^	3.62^b^
		21	6.28^b^	6.25^b^	1.72^c^	1.97^b^	0.15^b^	0.15^c^	3.12^b^	2.88^b^
		28	5.57^b^	5.68^b^	1.68^c^	1.86^b^	0.14^b^	0.15^c^	2.45^c^	2.67^b^

Year (Y)			*∗∗*	*∗∗*	*∗∗*	*∗∗*	*∗*	*∗*	*∗∗*	*∗∗*
Variety (V)			*∗∗*	*∗∗*	*∗∗*	*∗∗*	*∗*	*∗*	*∗∗*	*∗∗*
Year (Y) × variety (V)			*∗∗*	*∗∗*	*∗∗*	*∗∗*	*∗*	*∗∗*	*∗∗*	*∗∗*
Y × DAPA			*∗∗*	*∗∗*	*∗∗*	*∗∗*	*∗∗*	*∗∗*	*∗∗*	*∗∗*
V × DAPA			*∗∗*	*∗∗*	*∗∗*	*∗∗*	*∗∗*	*∗∗*	*∗∗*	*∗∗*
Y × V × DAPA			*∗∗*	*∗∗*	*∗∗*	*∗∗*	*∗∗*	*∗∗*	*∗∗*	*∗∗*

*∗* indicates statistical significance of the correlation coefficients at *p* < 0.05; *∗∗* indicates statistical significance of the correlation coefficients at *p* < 0.01; the mean values in a column with different letters indicate significant differences at *p* < 0.05 between treatment groups according to Duncan's multiple range test.
